# Object-based attention requires monocular visual pathways

**DOI:** 10.3758/s13423-024-02467-7

**Published:** 2024-02-13

**Authors:** N. Strommer, S. Al-Janabi, A. S. Greenberg, S. Gabay

**Affiliations:** 1 Department of Psychology, University of Haifa, Mount Carmel, 31905 Haifa, Israel; 2 The Institute of Information Processing and Decision Making (IIPDM), Haifa, Israel; 3 Department of Psychology, Charles Darwin University, Darwin, Northern Territory Australia; 4 Department of Biomedical Engineering, Medical College of Wisconsin & Marquette University, Milwaukee, WI USA

**Keywords:** Object-based attention, Neurocognition, Subcortical regions, Visual attention;

## Abstract

Mechanisms of object-based attention (OBA) are commonly associated with the cerebral cortex. However, less is known about the involvement of subcortical visual pathways in these processes. Knowledge of the neural mechanisms subserving OBA can provide insight into the evolutionary trajectory of attentional selection. In the current study, the classic double-rectangle cueing task was implemented using a stereoscope in order to differentiate between the involvement of lower (monocular) and higher (binocular) visual pathways in OBA processes. We found that monocular visual pathways are involved in two main aspects of OBA: exogenous orienting towards a cued object (Experiment 1; *N* =33) and attentional deployment within a cued object (Experiment 2; *N* =23); this is evident by the presence of OBA only when both the cue and target were presented to the same eye. Thus, these results indicate that monocular (mostly subcortical) visual regions are not simply passing information to higher cortical areas but have a functional computational role in OBA. These findings emphasize the importance of lower regions in attentional processes and, more specifically, in OBA.

The representational basis of attentional selection can be constructed upon spatial locations (space-based attention; SBA), objects (object-based attention; OBA), or other units (Egeth & Yantis, 1997). SBA involves attentional selection of a specific location in space. In SBA, attention is often considered to be a metaphoric spotlight that illuminates a region in space. Regions that fall within this spotlight are enhanced and afforded prioritized processing over those outside of it (Eriksen & Eriksen, 1974; Logan, 1996; Posner, 1980; Treisman & Gormican, 1988). In contrast, OBA involves selection of a specific object and is thought to encompasses all of the features associated with it (Kahneman & Henik, 1981; Neisser, 2014; Soto & Blanco, 2004), even when targets outside the object are closer in space (Greenberg et al., 2015) or participants attend overtly (Senturk et al., 2016). The double-rectangle cueing paradigm (Egly et al., 1994) has been used to simultaneously measure both SBA and OBA processes. In this detection task, two parallel rectangles are presented to participants. After fixation, an informative spatial cue appears, indicating the target’s location. The target can appear in one of three locations: the exact location predicted by the cue (valid condition), the far end of the cued object (invalid-same-object condition) or an equidistant location on the uncued object (invalid-different-object condition). As expected in attentional cueing experiments, Egly et al. (1994) observed faster responses on valid-cue trials than invalid-cue trials; an instantiation of SBA, as it indicates that the cue drew attention to its location, thereby facilitating target processing. For invalid-cue trials, responses were faster for the same-object condition than for the different-object condition, indicating an instantiation of OBA often termed a ‘same-object advantage’ (Chen, 2012; Müller, 2014).

The prevailing view of visual attention suggests that attention is guided by a cortical network of frontoparietal regions (Corbetta & Shulman, 2002; Kastner & Ungerleider, 2001; Pooresmaeili et al., 2014; Yantis & Serences, 2003), which provide biasing signals to the visual cortex, indicating which stimuli should be attended and which should be ignored (Desimone & Duncan, 1995; Greenberg et al., 2010, 2012). Object perception is also considered to be a cortical function, localized to the lateral occipital cortex (LOC; Grill-Spector, 2003; Serre et al., 2007). Moreover, there are evidences that the neural mechanism of OBA involves cortical areas such as the lateral occipital cortex, parietal and frontal regions, prefrontal cortex, inferior frontal junction, posterior parietal cortex, and right frontopolar cortex (Güldener et al., 2022; Kourtzi & Kanwisher, 2001; Malach et al., 1995; Müller & Kleinschmidt, 2003; Shomstein & Behrmann, 2006). Recently,

Cavanagh et al. (2023) proposed an innovative conceptual framework for OBA architecture. They posit that the frontoparietal attention networks selectively activate a particular object representation within the cortex’s object areas, contingent upon task relevance or expectation. Alternatively, the activation of the object regions can occur directly through bottom-up salience or cueing mechanisms intrinsic to the object. Once the target object representation is activated, it transitions into a preattentive object representation. Consequently, the object becomes the attentional target, whereby cascading top-down projections are deployed to facilitate its processing (Silver et al., 2007). These descending projections originating from the object representation extend to early visual cortices and the lateral geniculate nucleus (LGN), endowing the object with an essential amplification that enhances its processing.

In contrast to the cortical view of attention, attentional processes are adaptive for survival and are shared across many different species, some of which lack cortical tissue (Krauzlis et al., 2018). One example comes from studies that examined the existence of spatial orienting of attention in the archerfish (Gabay et al., 2013; Saban et al., 2017). The archerfish have an optic tectum but lack fully developed cortical structures. In these studies, archerfish were trained to perform a Posner cuing task (Posner et al., 1980) and demonstrated the typical pattern of results observed in humans—namely, both facilitation of target processing at a cued location during short cue-target delays; and inhibition of return (IOR) to that cued location during longer cue-target delays. These findings indicate that SBA can be performed without cortical involvement in certain species and, therefore, may rely upon both cortical and subcortical visual pathways.

The idea of subcortical involvement in attentional orienting in humans is not new. Gabay and Behrmann (2014) demonstrated that subcortical regions, specifically monocular visual channels, play a role in exogenous facilitation. They used a Posner task with a nonpredictive cue presented via a stereoscope, allowing segregation of monocular (subcortical) and binocular (cortical) pathways. They found that IOR occurred in both monoptic and dichoptic conditions, while exogenous facilitation was only observed in the monoptic condition. Their main finding suggests that subcortical structures are primarily involved in exogenous facilitation and potentially in attentional orienting.

Similarly to SBA, many assume that subcortical mechanisms are also involved in OBA processes, yet the empirical evidence for this claim is sparse. One example comes from an fMRI study that examined the neural substrates of both processes (Arrington et al., 2000). Participants’ performance was measured in a variant of the Posner spatial cueing task measuring both space-based and object-based cueing. The results revealed that both cortical areas (superior frontal gyrus, medial frontal gyrus, extrastriate regions, parahippocampal gyrus) and subcortical areas (pulvinar nucleus and inferior cerebellum) exhibited more activation for OBA than SBA. These results show that subcortical regions are implicated in OBA, as they are in SBA, but do not indicate that they are *necessary* for OBA, due to the correlational nature of neuroimaging.

The current study focuses on explicating the functional role of monocular (mostly subcortical) visual structures in OBA. In order to investigate the involvement of monocularly segregated subcortical regions of the visual processing stream, we employed a stereoscope. This apparatus enabled us to manipulate the visual information presented to each eye separately (Fig. 1), thereby allowing for the inference of causality regarding their functions for OBA. That is, this methodology affords us the capability to discern contributions emanating from monocular and binocular visual pathways, construed as subcortical and cortical contributions, respectively. Nonetheless, given the presence of monocular organization within V1 (as detailed below), in this paper, we will refer to involvement of monocular visual pathways at the level of V1 or earlier.

**Fig. 1:**
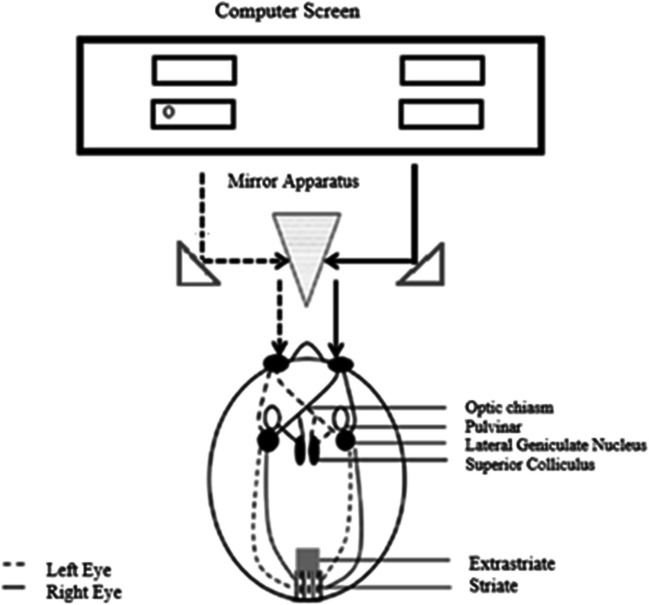
Schematic of the experimental apparatus and visual pathways. Each lens provided visual information to a different eye. Visual information passes through monocularly segregated subcortical regions (dashed lines left eye, solid lines right eye), is then projected to the lateral geniculate nucleus (LGN), and subsequently reaches striate and binocular extrastriate regions

## Experiment 1

**Fig. 2:**
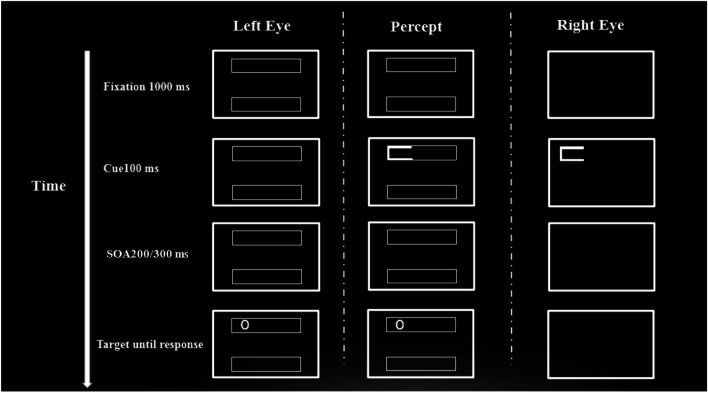
Example of a typical trial in Experiment 1. The figure depicts a valid object, dichoptic condition trial. The left column represents the presentation to the left eye, the right column represents the presentation to the right eye, and the center column represents the participants’ percept

The purpose of Experiment 1 was to investigate the attentional prioritization of a cued (versus uncued) object when the cue is segregated from the objects and targets (see Fig. 2). Isolating the involvement of monocular channels in the process of exogenous orienting towards an object would enable us to identify whether or not prioritization of an object by a spatial cue involves subcortical areas, or at least monocular visual pathways at the level of V1 or lower. That is, if OBA effect will be observed only in the monoptic condition, it will indicate that visual information regarding the cue and the object must be channeled through the same monocular pathway in order to elicit OBA. This will implicate monocular portions of the visual system and demonstrate their importance for this process.

### General method

The stereoscope method leverages the known monocular segregation of visual input through cortical area V1 (Menon et al., 1997). As visual information travels from the retina, through the LGN of the thalamus, terminating in the striate cortex (V1), the information is segregated monocularly; while neurons in the extrastriate cortex and beyond are primarily sensitive to binocular information (Byrne et al., 1997). Presenting different visual information (which must ultimately be integrated) to each eye separately is an effective method for isolating the involvement of monocular (mostly subcortical) neural channels. Hence, if subcortical (i.e., V1 and lower) areas are *functionally* involved in a process operating on the integrated percept then dividing the visual information by eye (i.e., segregating between the two monocular channels) should impair performance. In contrast, if subcortical areas are *not functionally* involved in a process, segregating the visual information between the eyes should not impair performance.

In two experiments, we aimed to understand the subcortical involvement in two aspects of OBA. First, we investigated the deployment of attention to an object as the result of a spatial cue. According to Cavanagh et al. (2023), the activation of an object can be done in a bottom-up manner. In this experiment, we wanted to examine whether this object’s facilitation is guided by monocular portions of the visual system. Specifically, we examined whether or not the prioritization of an object involves lower monocular channels. Here, we tested whether OBA is deployed only when both the cue and the object are presented to the same monocular channel. Secondly, we examined the influence of attentional deployment on the detection of a target within a cued object. That is, whether after the object is facilitated, does the processing of visual information presented within the object is guided by some subcortical mechanisms. Here, we tested whether OBA is deployed only when both the cued object and target are presented to the same monocular channel. To preview our results, we find that monocular (mostly subcortical) visual areas are necessary for both of these aspects of OBA.

### Method

#### Participants

Previous research that used the OBA task employed in the present study (Egly et al., 1994) revealed a very large effect size (η_p_^2^ = 0.51). However, because no previous study used the presentation manipulation employed in the current study, we erred on the side of caution in predicting only a medium to large effect size (η_p_^2^ = 0.08) to test within-subjects’ interactions. A power analysis (calculated using G*Power software; Faul et al., 2007) indicates that in order to detect within-group interaction effects, a total sample of 33 participants is needed to obtain statistical power at a 0.80 level with an alpha of 0.05. Therefore, a total sample of 33 participants (11 males; 24.8 ± 3.9 years) was used in the current study. All participants had normal or corrected-to-normal visual acuity (i.e., only corrective lenses, no spectacles). Participants were reimbursed for their time either via payment (30 NIS) or course credit. The Institutional Review Board (IRB) of the University of Haifa approved the experimental protocol.

#### Stimuli

The viewing distance was 45 cm. All stimuli were presented on a black background using a 22-in. CRT monitor with a 1,680 × 1,050 screen resolution and a 59-Hz refresh rate. Two rectangles formed the objects. Each rectangle measured 2.27° high × 11.48° wide and was oriented either vertically or horizontally in a block design. The rectangle frame was rendered in white, with a line thickness 0.5°. The cues were introduced by increasing the width of the object’s frame at the cued location from 0.5° to 1.1°. The target was the letter ‘O’ or ‘Q’ (Ariel Bold font, 0.501°) rendered in white. The screen was divided into two parts; each half of the screen was presented to a different eye through the use of a stereoscope (Fig. 2). On each side of the screen a large rectangle was presented to one eye (and was also used to enhance convergence between the eyes). All visual stimuli were presented within the large rectangular frames.

#### Procedure

Each trial began with the appearance of two parallel objects. Objects were oriented either horizontally or vertically in different blocks, and the order of blocks was counterbalanced between participants. This initial screen lasted for 1,000 ms, and was followed by a peripheral cue for 100 ms. After cue presentation, two possible SOAs were employed. One is of 300 ms and is similar to the one used by Egly et al. (1994). As a previous study (Gabay & Behrmann, 2014) demonstrated that the attentional time course might be different between monoptic and dichoptic presentations we also employed a 200-ms SOA. Hence, the target was presented after a variable stimulus onset asynchrony (SOA) of 200 or 300 ms in one of three possible locations: (1) the same location as the cue (i.e., valid condition), (2) the far end within the same object as the cue (i.e., invalid same object condition), or (3) the uncued object equidistant to the cue (i.e., invalid different object condition). Targets were present until participants responded and RTs were recorded. The cue validity was 33%; that is to say, the exogenous cue was not predictive of the target location. We employed exogenous cues pursuant to the finding that exogenous, nonpredictive cues yield stronger object-based selection (Goldsmith & Yeari, 2003). Participants were instructed to respond according to the target’s identity by pressing either ‘p’ or ‘q’ (counterbalanced between participants) on a bilingual computer keyboard as quickly and accurately as possible. On half of the trials, the cue, target, and object were presented to a single eye (monoptic presentation). On the other half of trials, the cue was presented to one eye and the target and object to the other eye (dichoptic presentation; see Fig. 2). Importantly, the full display (cue, target, & objects) was never presented to the participants binocularly. The factors manipulated in this task were target eye presentation (monoptic, dichoptic), cue validity (valid, invalid same object, invalid different object), object orientation (horizontal, vertical), and SOA (200 ms, 300 ms). Participants preformed four experimental blocks. Each block contained 140 trials.

### Results

Trials in which participants responded incorrectly were discarded (less than 3%). For each participant, responses in which RTs were faster or slower than 2 standard deviations from the mean of all participants and experimental conditions were also discarded (4.9%). A four-way repeated-measures analysis of variance (ANOVA) was conducted on RT in the discrimination task, with eye presentation (monoptic, dichoptic), cue validity (valid, invalid same object, invalid different object), object orientation (horizontal, vertical), and SOA (200 ms, 300 ms) as factors. See the Appendix for all means and standard deviations.

All main effects were significant. We observed a main effect of object orientation, *F*(1, 32) = 28.75, *p* < .001, η_p_^2^ = .47; horizontal: *M* = 531.05 )17.12); vertical: *M* = 559.9 (19.45), and a main effect of SOA, *F*(1, 32) = 15.48, *p* < .001, η_p_^2^ = .33; 200: *M* = 548.93 (17.98); 300: *M* = 542.02 (18.33), which was qualified by a significant interaction between object orientation and SOA, *F*(1, 32) = 32.95, *p* < .001, η_p_^2^ = .51. Planned contrasts revealed that RTs for the 200-ms SOA were slower than for the 300-ms SOA only in the horizontal condition, *F*(1, 32) = 60.35, *p* < .001, η_p_^2^ = .64; while there was no difference in the vertical condition, *F*(1, 32) = .56, *n.s.* We also observed a significant main effect of cue validity, *F*(2, 64) = 77.47, *p* < .001, η_p_^2^ = .71; invalid different: *M* = 558.38 (19.22); invalid same: *M* = 558.1 (19.85); valid: *M* = 519.95(15.71), and eye presentation, *F*(1, 32) = 17.08, *p* < .001, η_p_^2^ = .35; dichoptic: *M* = 551.28 (18.03); monoptic: *M* = 539.67 (18.34).

**Fig. 3:**
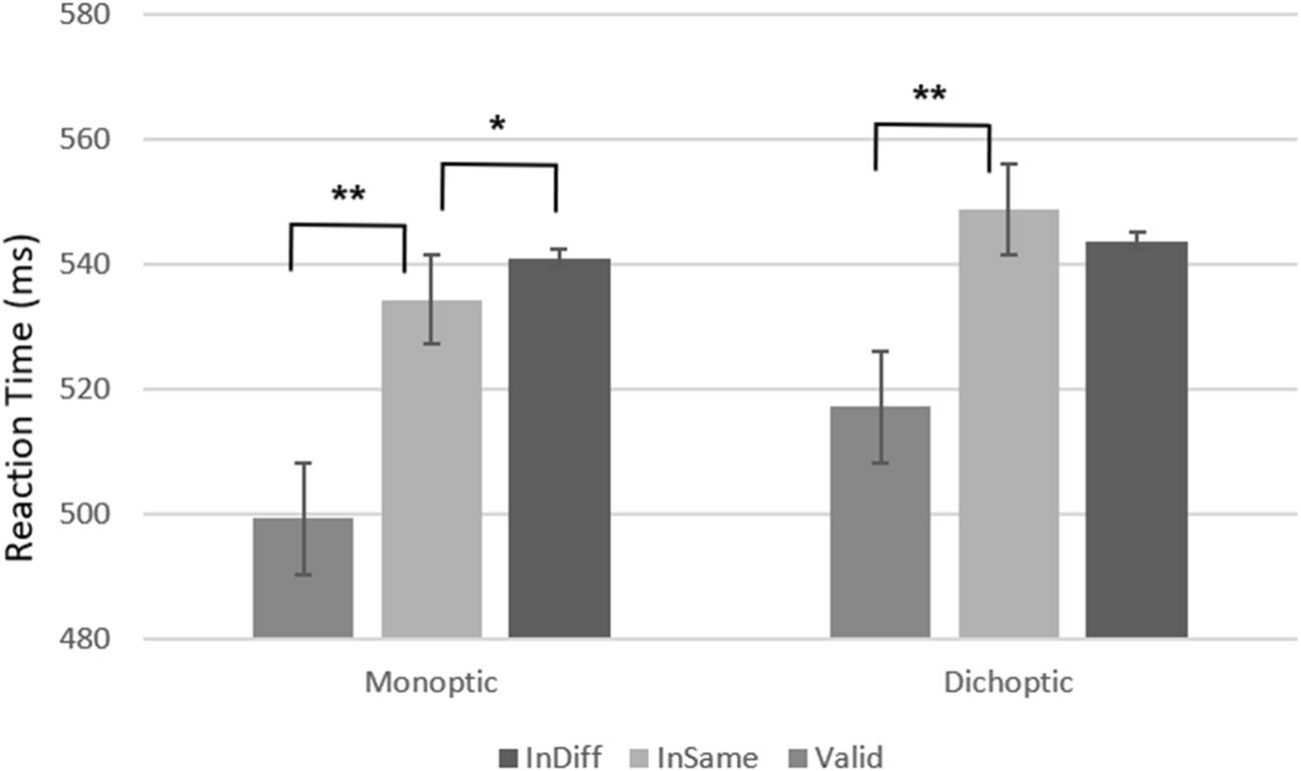
Experiment 1 results. The graph shows reaction time (ms) for eye presentation conditions as a function of cue validity. As can be seen, the object-based effect was significant only in the monoptic presentation condition. The values in the graph are collapsed across the different SOAs and orientation conditions. The error bars represent one standard error. *p < .05. **p < .01

Of primary importance, these main effects were qualified by a significant Cue Validity × Eye Presentation interaction, *F*(2, 64) = 5.01, *p* < .05, η_p_^2^ = .14. As can be seen in Fig. 3, planned contrasts revealed that the space-based effect (difference between valid and invalid same object condition) was significant, both in the monoptic presentation condition, *F*(1, 32) = 86.2, *p* < .001, η_p_^2^ = .68; valid: *M* = 499.34 (11.77), invalid same object: *M* = 534.34 (10.66), and in the dichoptic presentation condition, *F*(1, 32) = 38.93, *p* < .001, η_p_^2^ = .55; valid: *M* = 517.24 (10.32), invalid same object: *M* = 548.71 (12.17). In order to examine the OBA effect, we examined only the invalid conditions (invalid same and invalid different) between the two eye presentation conditions. This interaction was significant, *F*(1, 32) = 4.96, *p* < .05, η_p_^2^ = .13. Specifically, the object-based effect (difference between invalid same object and invalid different object conditions) was significant only in the monoptic presentation condition, *F*(1, 32) = 5.81, *p* < .05, η_p_^2^ = .15; invalid same object: *M* = 534.34 (10.66), invalid different object: *M* = 541.01 (11.72),^1^ and not in the dichoptic condition, *F*(1, 32) = 2.01, *n.s.*; invalid same object: *M* = 548.71 (12.17), invalid different object: *M* = 543.74 (10.5). No other interactions were significant (all *p*s > .05). These results indicate that object prioritization relies on subcortical areas.

Experiment 1 results showed that object-based attentional prioritization emerges only when the cue is presented to the same eye as the object and target. In contrast, we spatial attention prioritization was observed even if the cue was presented to a different eye. These results suggest a *necessary* functional role of subcortical areas in the deployment of OBA following an exogenous spatial cue.

## Experiment 2

**Fig. 4:**
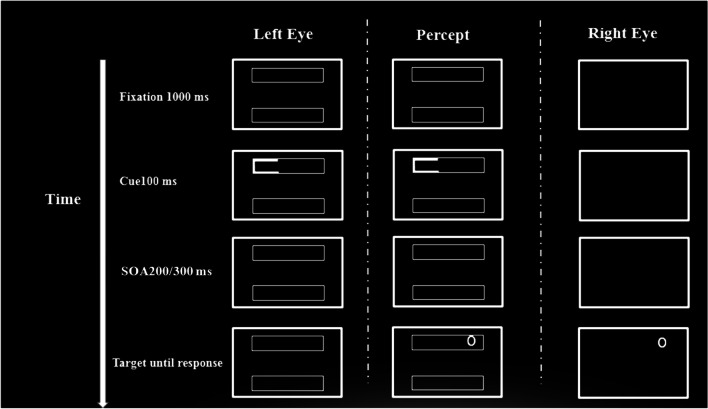
Example of a typical trial in Experiment 2. The figure depicts the invalid same object, dichoptic condition. The left column represents the presentation to the left eye, the right column is the presentation to the right eye, and the center column is the participants’ perception

The aim of the second experiment is to extend the prior findings by examining the involvement of subcortical areas in attentional deployment *within* objects (Fig. 4). If subcortical regions are functionally involved in attentional deployment within an object, we would expect that stereoscopically segregating the cue/object from the target would impair the prioritization of information within the cued object.

### Method

#### Participants

A power analysis based on the effect size observed in Experiment 1 (calculated using G*Power software; Faul et al., 2007) indicates that in order to detect within-group interaction effects, a total sample of 18 participants is needed to obtain statistical power at a 0.80 level with an alpha of 0.05. Therefore, a total sample of 23 participants (five males; 28.68 ± 4.2 years) was used in the current experiment. All participants had normal or corrected-to-normal visual acuity (i.e., only corrective lenses, no spectacles). Participants were reimbursed for their time either via payment (30 NIS) or course credit. The Institutional Review Board (IRB) of the University of Haifa approved the experimental protocol.

#### Stimuli

The stimuli were identical to those used in Experiment 1.

#### Procedure

The procedure was similar to that used in Experiment 1, with one exception: Rather than the cue being segregated stereoscopically, the target was presented now in either a monoptic or dichoptic presentation with the cue/object.

### Results

Trials in which participants responded incorrectly were discarded (less than 3%). For each participant, responses in which reaction times (RTs) were faster or slower than 2 standard deviations from the mean of all participants, and experimental conditions were also discarded (5.1%). A four-way repeated-measures ANOVA was conducted on RTs for correct trials as the dependent variable and eye presentation, cue validity, object orientation, and SOA as factors. See the Appendix for all means and standard deviations.

Three of the main effects were significant: a significant main effect of cue validity, *F*(2, 44) = 15.21, *p* < .001, η_p_^2^ = .41; invalid different: *M* = 580.85 (16.52); invalid same: *M* = 577.96 (17.21); valid: *M* = 553.12 (16.15)], a significant main effect of eye presentation, *F*(1, 22) = 20.75, *p* < .001, η_p_^2^ = .49; dichoptic: *M* = 587.8 (18.84); monoptic: *M* = 553.49 (14.36), and a significant main effect of object orientation, *F*(1, 22) = 4.92, *p* < .05, η_p_^2^ = .18; horizontal: *M* = 562.31 (16.15); vertical: *M* = 578.98 (17.32). These main effects were qualified by three interactions. The interaction between object orientation and cue validity, *F*(2, 44) = 7.63, *p* < .005, η_p_^2^ = .26, was qualified by a three-way interaction involving object orientation, eye presentation, and SOA, *F*(1, 22) = 5.1, *p* = .035, η_p_^2^ = .19. This interaction resulted from a smaller difference between the two eye presentation conditions at the first SOA compared with the second SOA in the horizontal object orientation condition, *F*(1, 22) = 6.49, *p* < .05, η_p_^2^ = 0.23. No such difference was observed in the vertical object orientation condition, *F*(1, 22) = 1.62, *n.s*.

**Fig. 5:**
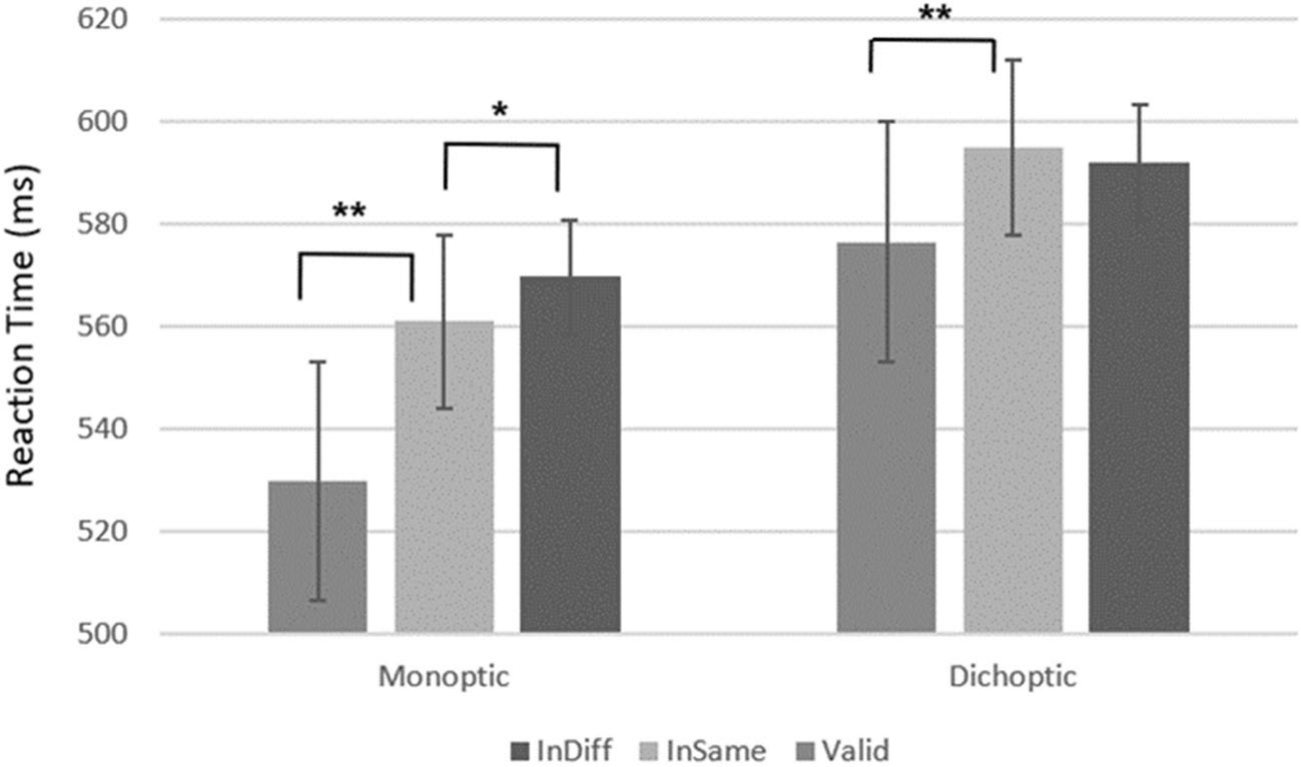
Results from Experiment 2. The graph shows reaction time (ms) for both eye presentation conditions as a function of cue validity. Results show that the object-based effect was significant only in the monoptic presentation condition. The values in the graph are collapsed across the different SOAs and orientation conditions. The error bars represent the standard error. *p < .05. **p < .01

Most importantly, as predicted, there was a significant interaction between cue validity and eye presentation, *F*(2, 44) = 3.87, *p* = .028, η_p_^2^ = .15. As can be seen in Fig. 5, planned contrasts revealed that the space-based effect was significant both in the monoptic condition, *F*(1, 22) = 27.39, *p* < .001, η_p_^2^ = .55, valid: *M* = 529.76 (11.89), invalid same object: *M*= 561.01 (15.08), and in the dichoptic condition, *F*(1, 22) = 5.1, *p* < .05, η_p_^2^ = .18; valid: *M* = 576.47 (21.31), invalid same object: *M* = 594.91 (19.72). In order to examine the OBA effect, we examined only the invalid conditions (Invalid Same and Invalid Different) between the two Eye Presentation conditions. This interaction was not significant, *F*(1, 22) = 2.38, *p* = .13. Nevertheless, when examining the object-based effect, it was significant only in the monoptic condition, *F*(1, 22) = 5.38, *p* < .05, η_p_^2^ =.19; invalid same object: *M* = 561.01 (15.08), invalid different object: *M* = 569.7 (16.9),^2^ and not dichoptic condition, *F*(1, 22) = .31, *n.s.*; invalid same object: *M* = 594.91 (19.72), invalid different object: *M* = 592.02 (16.8).^3^ No other effects were significant (all *p*s > .05).

Experiment 2 results demonstrated that object-based attentional prioritization occurs only when the target is presented to the same eye as the cue and the object. In contrast, spatial attention prioritization was evident even if the target was presented to a different eye. These results replicate the findings of the first experiment and demonstrate *the* functional role of monocular (mostly subcortical) pathways in the deployment within objects.

It is important to note there are differences in the general RTs between the two experiments. Those differences might result from individual differences, as different groups of participants were tested in the different experiments. Consequently, variations in reaction times could be attributed to individual differences among the subjects. However, it is essential to note that in each experiment, the analysis employed a within-subject design, mitigating the potential impact of individual differences and allowing for the identification of effects within each group.

## General discussion

The present study provides new insights regarding the neural substrates of OBA. In two experiments, we demonstrated the necessity of lower visual pathways in attentional deployment both towards and within an object. In Experiment 1, attentional deployment towards an object (following an exogenous spatial cue) depended on monocular visual channels, as evidenced by the monocular advantage effect. In Experiment 2, attentional deployment within an object (following appearance of the target) depended on monocular channels, as evidenced by the monocular advantage effect.

Recently, a vast review by Cavanagh et al. (2023) proposed a framework for attentional deployment in which frontoparietal attention networks activate a specific object representation in the object areas of the cortex, either via top-down processes based on task relevance or expectation, or through bottom-up salience or cueing. Once activated, it becomes preattentive object representation and serves as the target for attention, with downward projections facilitating its processing. In contrast to this proposed framework, our findings highlight the involvement of lower visual pathways in attentional deployment towards and within an object. Specifically, our experiments demonstrated the importance of some subcortical areas, particularly the reliance on monocular visual channels, for attentional deployment, and challenge the notion of activation solely within the cortical regions as suggested (Cavanagh et al., 2023). Although the current study cannot dissociate the involvement of early cortical visual areas (e.g., V1) from subcortical mechanisms, it was previously suggested that subcortical structures are involved in the conscious processing of objects, specifically the lateral geniculate nucleus (LGN; Levinson et al., 2021). Additionally, a subcortical magnocellular route that passes through the superior colliculus (SC) and the pulvinar is involved in the fast processing of objects (Wang et al., 2023). These findings strengthen the claim that subcortical regions do play a role in OBA. Considering these findings collectively, it is conceivable to assert that V1 or even lower regions do not merely serve as conduits for transmitting information to higher cortical regions, but have a functional significance in OBA. Drawing from earlier research that suggested the involvement of subcortical regions in attentional processes (specifically, the pulvinar and SC; Gabay & Behrmann, 2014; Gattass et al., 2018; Guedj & Vuilleumier, 2023; Wang et al., 2023), we interpret the present findings as further evidence supporting the role of subcortical structures in attentional mechanisms. However, further investigations are warranted to delve into the specific neural mechanisms underlying these processes.

The divergence between our study and existing literature highlights the importance of including subcortical regions in attentional process research. Recognizing their contribution enhances our understanding of the neural substrates underlying OBA. Our findings support an evolutionary framework, suggesting monocular engagement in attentional processes as an adaptive mechanism. It is worth noting that our results don’t invalidate the role of cortical regions; instead, they offer a more nuanced and inclusive perspective on attentional control mechanisms. Future research should investigate the dynamic interplay between cortical and subcortical regions to gain deeper insights into the complex neural networks involved in object-based attention.

Moreover, the involvement of lower visual mechanisms in OBA might not be surprising when considering the similar (to humans) benefit that less evolved organisms (e.g., archerfish) would gain from prioritizing objects when scanning the visual field. Object perception has previously been demonstrated in lower order species, such as pigeons (Cook et al., 2001) and fish (von der Emde, 2006). OBA may, therefore, be an adaptive and strategic process (Greenberg & Gmiendl, 2008; Shomstein, 2012), and, as such, might also be evident in other less evolved species.

In the current study, a modulation of OBA, but not SBA, as a function of eye presentation was observed. This dissociation might be explained by the specific SOAs used in this study. As noted, Gabay and Behrmann (2014) showed that exogenous facilitation is delayed when the cue and target are presented dichoptically. Interestingly, in the dichoptic presentation condition, facilitation was not apparent in the earlier SOAs (e.g., 100 ms) but did emerge in the later SOAs (e.g., 225 ms). In the monocular condition, facilitation was apparent even in the earlier SOAs (e.g., 100 ms). In the current study, the shortest SOA was 200 ms, which was long enough for SBA to manifest even when the cue and target were presented dichoptically but not long enough for OBA to manifest under such conditions. Although facilitation was observed and was significant in both eye presentation conditions, the pattern of results showed a smaller facilitation in the dichoptic condition compared to the monoptic condition (although this pattern was not statistically significant, it was present in both experiments). Perhaps this result indicates a difference in the time course of OBA and SBA in recruiting the involvement of subcortical areas.

To summarize, the experiments reported in this study support the notion that OBA processes recruit lower monocular pathways, as demonstrated here for the first time (to our knowledge). Notable observation occurs is the prevalent tendency within cognitive neuroscience to predominantly investigate and associate cognitive processes with cortical regions. By elucidating the involvement of these lower neural areas in OBA processes, this study challenges the prevailing paradigm and broadens our perspective on the neural underpinnings of cognition. It serves as a clarion call to reevaluate and expand our research focus, enabling a more holistic understanding of cognitive processes that extends beyond the boundaries of solely cortical regions. The findings presented in this study pave the way for further investigations into the intricate interplay between cortical and subcortical visual regions in supporting cognitive functions. By acknowledging the significance of V1 and lower monocular pathways contributions to OBA processes, we can unlock new avenues for exploring the neural dynamics that shape attentional mechanisms and inform our comprehension of higher-order cognitive abilities.

In conclusion, this study not only establishes the involvement of lower visual regions in OBA processes but also emphasizes the necessity to transcend the cortical-centric approach prevalent in cognitive neuroscience. By broadening our horizons and embracing a more inclusive perspective, we can achieve a more comprehensive and accurate understanding of the neural basis of cognition.

## Data Availability

All data will be provided by request.

## Appendix

Means and standard deviation (*SD*) for each condition

Experiment 1

**Table:**

Within-subjects factors								
Orientation		SOA	Eye presentation		Cue validity		Mean	*SD*
	Horizontal	200		Dichoptic		Invalid diff	534.69	58.94
						Invalid same	542.70	70.56
						Valid	514.74	57.74
				Monoptic		Invalid diff	534.32	70.29
						Invalid same	525.52	55.01
						Valid	500.61	66.10
		300		Dichoptic		Invalid diff	521.31	65.55
						Invalid same	521.58	75.69
						Valid	495.25	57.02
				Monoptic		Invalid diff	522.25	64.60
						Invalid same	514.11	67.72
						Valid	478.47	62.35
	Vertical	200		Dichoptic		Invalid diff	554.70	65.29
						Invalid same	569.93	87.76
						Valid	524.71	66.23
				Monoptic		Invalid diff	550.70	78.13
						Invalid same	545.59	75.22
						Valid	514.56	82.31
		300		Dichoptic		Invalid diff	564.27	71.17
						Invalid same	560.65	74.75
						Valid	534.26	75.13
				Monoptic		Invalid diff	556.79	77.36
						Invalid same	552.16	71.10
						Valid	503.74	75.91

Experiment 2

**Table:**

Within-subjects factors								
Orientation		SOA	Eye presentation		Cue validity		Mean	*SD*
	Horizontal	200		Dichoptic		Invalid diff	591.77	83.29
						Invalid same	570.39	76.93
						Valid	553.66	77.19
				Monoptic		Invalid diff	588.45	130.32
						Invalid same	551.38	83.53
						Valid	535.58	67.54
		300		Dichoptic		Invalid diff	577.50	78.32
						Invalid same	584.51	111.63
						Valid	577.57	125.52
				Monoptic		Invalid diff	560.21	79.12
						Invalid same	538.84	79.23
						Valid	517.90	56.86
	Vertical	200		Dichoptic		Invalid diff	599.59	92.08
						Invalid same	604.60	93.64
						Valid	596.96	140.59
				Monoptic		Invalid diff	559.89	65.36
						Invalid same	559.18	69.01
						Valid	536.51	60.73
		300		Dichoptic		Invalid diff	599.19	94.04
						Invalid same	620.17	129.09
						Valid	577.73	127.57
				Monoptic		Invalid diff	570.27	81.27
						Invalid same	594.67	87.06
						Valid	529.08	69.48

## Footnotes

1 Note that this effect was also significant when RT was normalized for the differences between the two presentation conditions by using the mean RT for each participant at each presentation condition as baseline (RTnormalized = RTunnormalized − RTmean).

2 Note that this effect was also significant when RT was normalized for the differences between the two presentation conditions by using the mean RT for each participant at each presentation condition as baseline (RTnormalized = RTunnormalized − RTmean).

3 This pattern was also observed when examining only the horizontal condition.
